# The Characterization of Fixation of Ba, Pb, and Cu in Alkali-Activated Fly Ash/Blast Furnace Slag Matrix

**DOI:** 10.3390/ma9070533

**Published:** 2016-06-30

**Authors:** Jan Koplík, Lukáš Kalina, Jiří Másilko, František Šoukal

**Affiliations:** Materials Research Centre, Faculty of Chemistry, Brno University of Technology, Purkyňova 118, Brno 61200, Czech Republic; kalina@fch.vut.cz (L.K.); masilko@fch.vut.cz (J.M.); soukal@fch.vut.cz (F.Š.)

**Keywords:** fixation, alkali-activated materials, X-ray photoelectron spectroscopy, heavy metals

## Abstract

The fixation of heavy metals (Ba, Cu, Pb) in an alkali-activated matrix was investigated. The matrix consisted of fly ash and blast furnace slag (BFS). The mixture of NaOH and Na-silicate was used as alkaline activator. Three analytical techniques were used to describe the fixation of heavy metals—X-ray photoelectron spectroscopy (XPS), scanning electron microscopy (SEM) equipped with energy dispersive X-ray spectroscopy (EDS), and X-ray powder diffraction (XRD). All heavy metals formed insoluble salts after alkaline activation. Ba was fixed as BaSO_4_, and only this product was crystalline. EDS mapping showed that Ba was cumulated in some regions and formed clusters. Pb was present in the form of Pb(OH)_2_ and was dispersed throughout the matrix on the edges of BFS grains. Cu was fixed as Cu(OH)_2_ and also was cumulated in some regions and formed clusters. Cu was present in two different chemical states; apart from Cu(OH)_2_, a Cu–O bond was also identified.

## 1. Introduction

Heavy metals are a significant part of various industrial and chemical waste materials, which can be a serious environmental threat. Waste materials with heavy metal content are processed by the set of procedures called stabilization/solidification. During these procedures, many different materials such as concrete and glass are used. In the past few decades, a new material for the inhibition of hazardous materials was used—alkali-activated material (AAM) [1,2].

The history of AAMs began in 1940s, when Kühl [3] and later Purdon [4] used an alkali activator for the activation of blast furnace slag. Nowadays, AAMs include broad types of materials—blast furnace slag, metakaolin, fly ash, etc. However, AAMs can vary in chemical or phase composition, and an alkali activator (high pH) is always used during the preparation of them. The main difference among AAMs is the content of calcium. AAMs can be divided into the groups of high-calcium alkali-activated materials (HCAAMs) and low-calcium alkali-activated materials (LCAAMs). HCAAMs are represented by alkali-activated blast furnace slag (BFS) or other types of slag (steel, phosphorus, copper, nickel, etc.). The C–S–H and C–A–S–H gels are assumed to be the main hydration products of alkali activation of HCAAMs. This gel has a similar structure like disordered tobermorite-like C–S–H(I) type and AlO_4_ tetrahedra enable the extension or crosslinking of the silicon chains. The secondary hydration products are formed depending on the composition of blast furnace slag and used activator. AFm type phases were identified when using NaOH as the activator, hydrotalcite was identified in activated slags with a high amount of MgO, and zeolites were identified in BFS with a high amount of Al_2_O_3_ [5,6,7,8,9,10]. A considerably different structure is present after the alkali activation of LCAAMs. The structure can be described as a disordered cross-linked aluminosilicate network, which consists of SiO_4_ and AlO_4_ tetrahedra linked by oxygen atoms. The negative charge of AlO_4_ is balanced by the presence of a positive ion in the structure. Usually Na^+^ or K^+^ serve as the charge-balancing ions, but Ca^2+^, Ba^2+^, Mg^2+^, and NH^4+^ can also occur. In fact, the structure of LCAAMs is very similar to the structure of zeolites [5,11,12]. The main reaction product of the alkali activation of the mixture of both types of AAMs (BFS and fly ash) is the N–A–S–H type gel. The gel is actually of a C–S–H type with a high concentration of tetrahedrally coordinated Al and an interlayer of Na ions incorporated into its structure [13].

The ability of AAMs to immobilize heavy metals and other hazardous materials in their structure has been investigated since the second half of the last century. The efficiency of the inhibition of heavy metals and other hazardous materials within AAMs depends on more factors including the type of immobilized material, its dosage, the type of matrix, the nature of alkaline activator, and its concentration. In general, Pb, Zn, Cr, and B reach a high efficiency of immobilization in AAMs, as opposed to Cu, Cd, and As, which have worse inhibition results. There are two different ways of how to fix heavy metals in the matrix—physical and chemical. Often, both of these types occur at the same time. The physical immobilization of heavy metals is the same in AAMs as in other binders (e.g., ordinary Portland cement). The physical immobilization is linked with the mechanical properties of matrix and its porosity. The chemical fixation means bonding with newly formed alumino-silicate phases. Some metals are fixed by forming insoluble salts, mainly hydroxides or silicates [14,15,16,17,18,19,20,21,22,23,24,25,26,27,28,29]. 

The aim of this study was to describe, how heavy metals (Ba, Pb, Cu) are fixed within the matrix based on the alkali-activated mixture of fly ash and BFS, what their chemical state is, and how they are distributed in the matrix. The description of form and chemical state of heavy metals after the activation was enabled by using a modern analytical method—X-ray photoelectron spectroscopy.

## 2. Materials and Methods

### 2.1. Sample Preparation 

Blast furnace slag and high-temperature fly ash were used as raw materials. Their chemical composition is shown in Table 1. The mixture of NaOH and Na-silicate (weight ratio SiO_2_:Na_2_O—3.1; wt % SiO_2_—27.3) was used as the alkaline activator. Heavy metals were used in the form of Ba(NO_3_)_2_ p. a., Pb(NO_3_)_2_ p. a., and CuCl_2_∙2H_2_O p. a. They were added as solids in the dosage of 2.5 wt % of binder. Finally, three matrices with added heavy metals were prepared—one matrix for each heavy metal. Demineralized water was used throughout. The composition of matrices is listed in Table 2. The preparation of the matrix started with the mixture of all solid compounds together for 5 min, followed by the adding of the alkaline activator and mixing for another 5 min. After mixing, the samples were cast in steel molds measuring 20 × 20 × 100 mm^3^ and vibrated for 90 s. All analyses were performed after 28 days of open curing at ambient conditions.

### 2.2. Methods

The X-ray photoelectron spectroscopy (XPS) analyses were carried out with an Axis Ultra DLD spectrometer (Kratos Analytical Ltd., Manchester, UK) using a monochromatic Al Kα (hν = 1486.7 eV) X-ray source operating at 150 W (10 mA, 15 kV). The spectra were obtained using the analysis area of ~300 × 700 μm. The Kratos charge neutralizer system was used for all analyses. The high-resolution spectra were measured with the step size of 0.1 eV and 20 eV pass energy. The instrument base pressure was 2 × 10^−8^ Pa. The spectra were analyzed using the CasaXPS software (version 2.3.15) (Casa Software Ltd., Teignmouth, UK) and were charge-corrected to the main line of the carbon C 1s spectral component (C–C, C–H) set to 284.80 eV. Standard Shirley background was used for all sample spectra. 

The scanning electron microscopy (SEM) and energy dispersive X-ray spectroscopy (EDS) analyses were performed using a JEOL JSM-7600F (JEOL Ltd., Tokyo, Japan) scanning electron microscope equipped with X-Max 80 mm^2^ SDD detector in back-scattering mode. The working distance for all samples was 15 mm, the accelerate voltage was 15 kV, and the probe current was 1 nA. Prior to the SEM analysis, all samples were polished for 6 h by a cross-section polisher JEOL (JEOL Ltd., Tokyo, Japan). All samples were sputtered by gold to obtain good surface conductivity. 

The X-ray powder diffraction (XRD) data were obtained using a PANanalytical Empyrean (PANanalytical B.V., Almelo, The Netherlands) diffractometer with CuKα radiation (1.54 Å) operating at the voltage of 40 kV, and the current of 30 mA and equipped with 3D detection system PIXcel3D (PANanalytical B.V., Almelo, The Netherlands). All samples were step-scanned from 5° to 90° 2θ using vertical high-resolution goniometer with a step size of 0.013° 2θ. Time per step was 96 s. The variable slits with an irradiated length of 10 mm were used. Presented X-ray patterns have an original background that has not been subtracted. 

## 3. Results and Discussion

### 3.1. XPS

**Figure 1:**
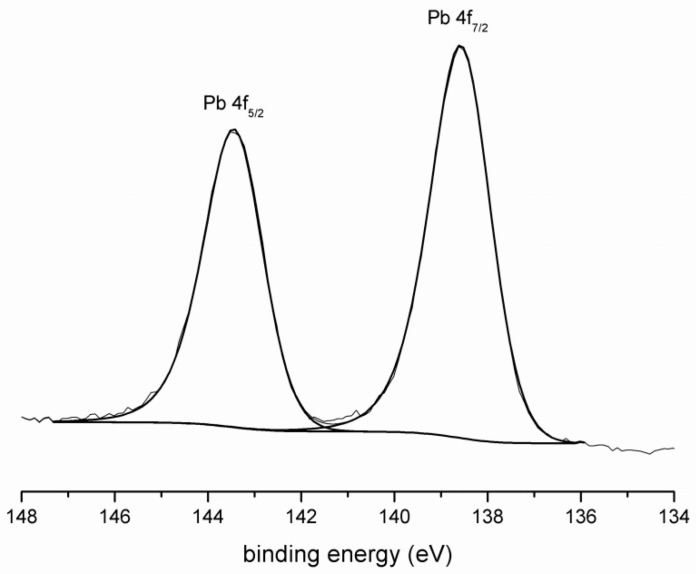
X-ray photoelectron spectroscopy (XPS) spectrum of Pb.

The XPS results show the chemical state of examined elements (Pb, Ba, Cu). In the case of lead, the high-resolution spectrum gives the binding energy of 138.6 eV for Pb 4f_7/2_ line (Figure 1), typical for Pb(OH)_2_. The conversion of primary phase Pb(NO_3_)_2_ to hydroxide will probably take place in the same way as in the hydration of Portland cement. This salt forms insoluble hydroxide in an alkaline solution and may form a coating on the cement (blast furnace slag) particles, which can be observed in Figure 6. There is no evidence that the lead compound creates highly insoluble Pb_3_SiO_5_ as in the system of alkali-activated fly ash in Palomo et al.’s study [29].

**Figure 2:**
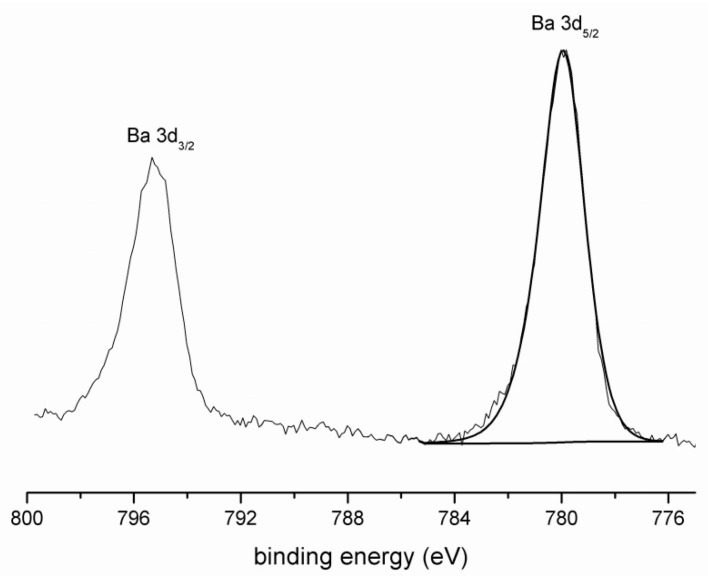
XPS spectrum of Ba.

The Ba 4d spectrum (Figure 2) demonstrates the creation of BaSO_4_ with a binding energy of Ba 3d_5/2_ of 780.0 eV, which is consistent with the EDS analysis. 

**Figure 3:**
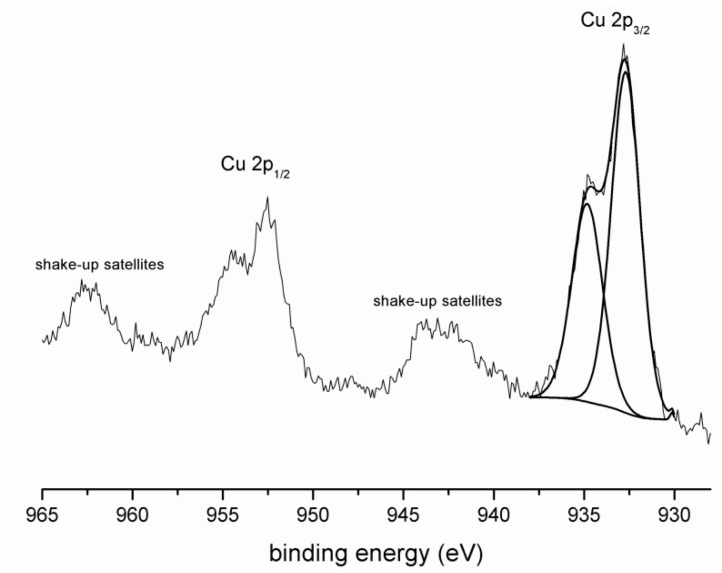
XPS spectrum of Cu.

Finally, the XPS spectrum of Cu 2p (Figure 3) shows two different chemical states. The first Cu 2p_3/2_ component at 934.8 eV with a strong shake-up satellite belongs to Cu(OH)_2_. Shake-up peaks may occur when the outgoing photoelectron simultaneously interacts with a valence electron and excites it to a higher-energy level. The kinetic energy of the shaken-up core electron is then slightly reduced giving the satellite structure a few eV below (higher on the calculated binding energy scale) the core level position [30]. It should be noted that the determination of Cu(OH)_2_ also depends on the peak shape and main peak to shake up peak separation. The second Cu 2p_3/2_ peak at 932.7 eV represents the bond of Cu with O. That can be interpreted as the formation of copper oxide or the formation of the bond with the matrix.

### 3.2. SEM

The microstructure of prepared matrices with heavy metals was characterized by the SEM analysis. The regions containing heavy metals were investigated with EDS analysis. In all images, remaining particles of fly ash and grains of BFS are evident. High-temperature fly ash consists of spherical particles of various sizes. Some of them can be hollow, and smaller particles are encapsulated in bigger ones in many cases. BFS is represented by grains of irregular shape. The remaining space is filled by alumino-silicate gel. 

**Figure 4:**
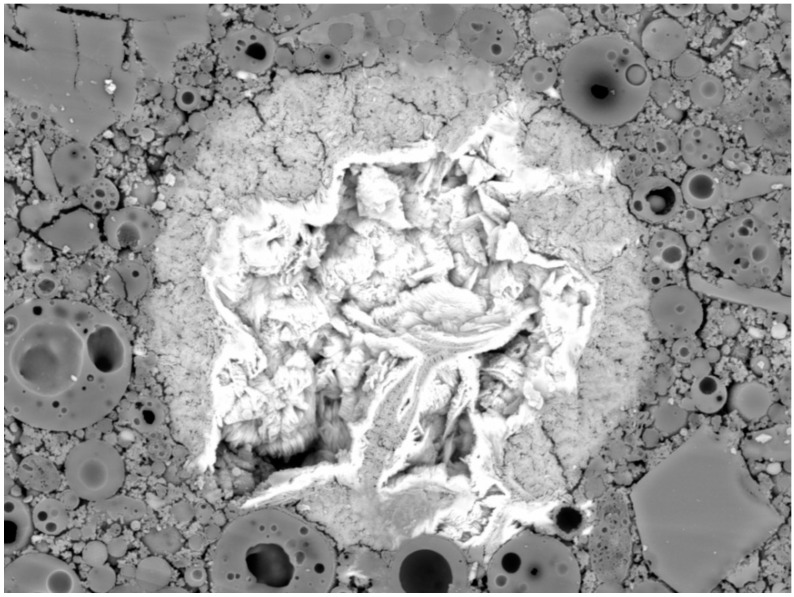
Scanning electron microscopy (SEM) image of the region with fixed barium, magnitude 2000×.

Figure 4 shows the SEM image of the prepared matrix. The light structure in the middle of the image represents the area with a high amount of barium. Performed EDS analysis determined barium, sulfur, and oxygen as the main components of the structure (Table 3). Their molar ratio of 1:1:4 responds to BaSO_4_. These results correspond with the XPS analysis. BaSO_4_ was also identified by the XRD analysis. Subsequently, the mapping of chemical elements within the matrix was performed. The mapping (Figure 7) showed that Ba cumulated in some bigger areas such as BaSO_4_, rather than being dispersed uniformly throughout the matrix. Unlike Cu and Pb, Ba did not form hydroxide because of the good solubility of Ba(OH)_2_. The sulfates originated from raw materials due to the fact that the chemical composition of both fly ash and blast furnace slag contained a sufficient amount of sulphur to form BaSO_4_. 

**Figure 5:**
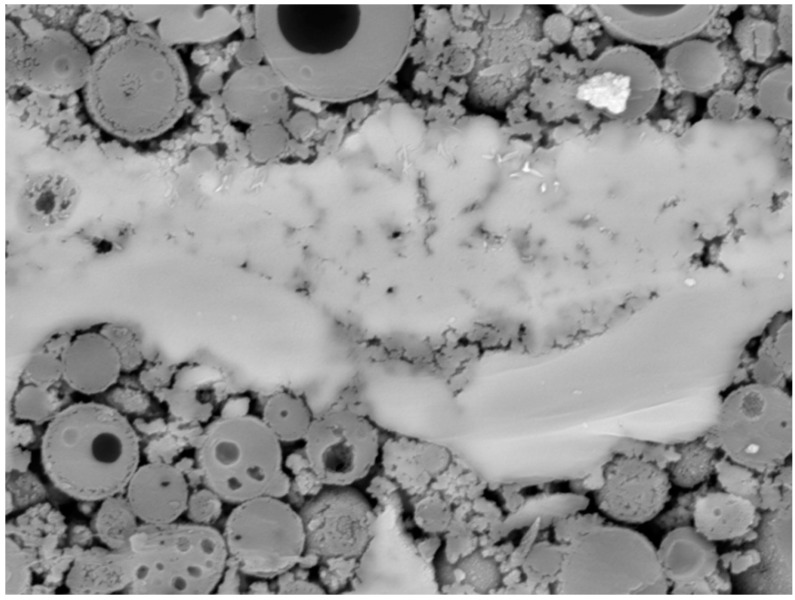
SEM image of region with fixed Cu, magnitude 7000×.

In Figure 5, the SEM image of the area with cumulated Cu is displayed. It can be seen as “foggy” lighter structure of irregular shape in the central part of image. The prevailing components are Cu and O (Table 4). The molar ratio of 1:1.6 shows Cu(OH)_2_ as a possible reaction product of Cu. This assumption was proved by the XPS analysis. No Cu(OH)_2_ was identified by the XRD analysis. It indicates a non-crystalline character of Cu(OH)_2_. The mapping showed similar results as in the case of barium. Cu tends to cumulate in small areas of Cu(OH)_2_ rather than being dispersed throughout the matrix. The difference of distribution between barium and copper is that copper forms a higher number of smaller clusters. 

**Figure 6:**
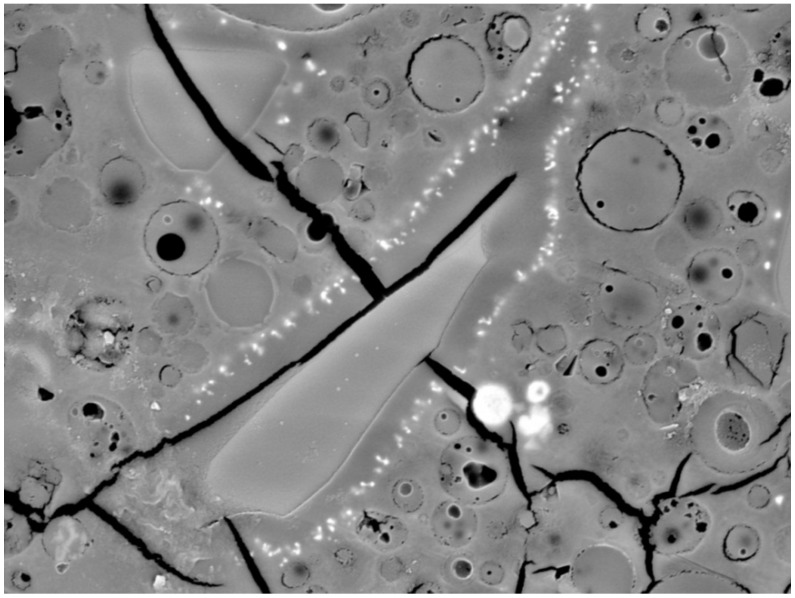
SEM image of Pb coating on the BFS grain, magnitude 4000×.

**Figure 7:**
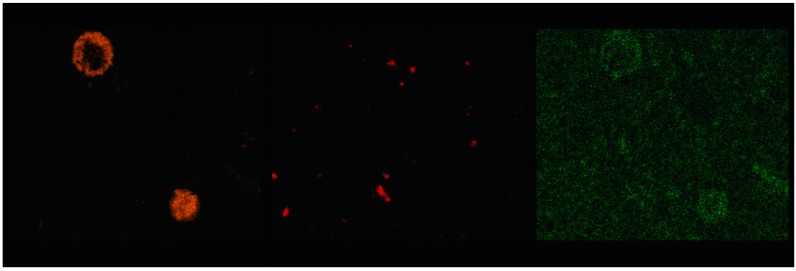
EDS mapping of Ba, Cu, and Pb in the matrix.

Figure 6 shows the detail of a grain of BFS. A coating of small particles are visible on the edges of the grain. The EDS analysis proved that the coating contained Pb (Table 5). The particles are supposed to be formed of Pb(OH)_2_ according to XPS analysis. Unlike both previous elements, Pb did not cumulate in larger clusters of Pb(OH)_2_, but it seems to be dispersed throughout the matrix (Figure 7). The reason that Pb did not form bigger clusters could be caused by the smaller amount of Pb atoms in the matrix. All metals were added in wt %, so the amount of Pb atoms in the matrix is the lowest, because of its superlative molar weight.

### 3.3. XRD

**Figure 8:**
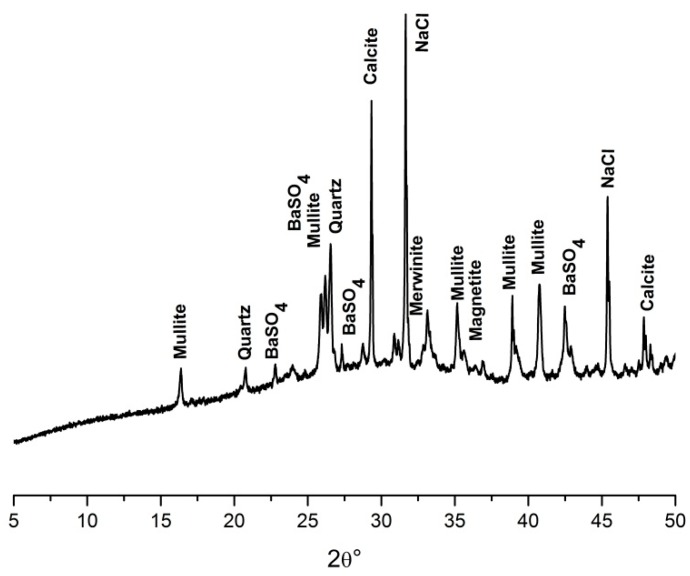
X-ray powder diffraction (XRD) patterns of the alkali-activated material (AAM) matrix with heavy metals.

The XRD analysis identified only one crystalline compound of fixed heavy metals—BaSO_4_. The XRD patterns of the alkali-activated matrix are shown in Figure 8. The patterns of BaSO_4_ are described, the rest of the patterns belongs to the phases of the matrix (quartz, mullite, merwinite, calcite, magnetite) and to NaCl. No compounds of Pb and Cu were identified by XRD, since they are fixed in the amorphous phases.

## 4. Conclusions

The fixation of three heavy metals—Ba, Pb, and Cu—in alkali-activated matrices based on blast furnace slag and fly ash was examined. The XPS analyses proved that all metals formed insoluble salts—BaSO_4_, Pb(OH)_2_ and Cu(OH)_2_—after alkaline activation. Only Cu was present in matrices in two different chemical states; apart from Cu(OH)_2_, a Cu–O bond was also identified. Both Pb(OH)_2_ and Cu(OH)_2_ were present in an amorphous or semicrystaline form, which could not be identified by XRD. The distribution of heavy metals within the matrices was quite different. Ba and Cu were not dispersed throughout the matrix evenly and were cumulated in some regions. On the other hand, EDS mapping showed that Pb did not form clusters and was present as a coating on the edges of grains of the blast furnace slag in the whole matrix. The results show that the metals were fixed mainly by encapsulation in the matrix and forming insoluble salts. Only in the case of Cu can the formation of some chemical bond with newly formed phases after alkali activation be assumed.

## Figures and Tables

**Table 1:** Chemical composition of fly ash and BFS (wt %).

**Fly Ash**									
SiO_2_	Al_2_O_3_	CaO	Na_2_O	K_2_O	MgO	SO_3_	Fe_2_O_3_	TiO_2_	P_2_O_5_
50.30	27.70	3.84	0.76	2.67	1.15	0.87	10.40	1.45	0.28
**Blast Furnace Slag**									
SiO_2_	Al_2_O_3_	CaO	Na_2_O	K_2_O	MgO	SO_3_	Fe_2_O_3_	TiO_2_	MnO
34.70	9.05	41.1	0.41	0.90	10.5	1.46	0.25	0.96	0.55

**Table 2:** Composition of matrices (wt %).

Slag	Fly ash	NaOH	Na_2_O.SiO_2_	Water	Ba, Pb, Cu
15.6	57.4	6.1	6.2	12.2	2.5

**Table 3:** Energy dispersive X-ray spectroscopy (EDS) analysis of the Ba cluster (atom %).

O	Na	Si	S	Cl	K	Ca	Fe	Ba
62.86	0.59	1.96	14.44	1.76	1.12	0.32	0.59	16.36

**Table 4:** EDS analysis of the Cu cluster (atom %)

O	Al	Si	Cl	Ca	Fe	Cu	Pb
55.8	0.87	6.91	1.31	0.34	0.28	33.79	0.38

**Table 5:** EDS analysis of the Pb coating (atom %).

O	Mg	Al	Si	S	K	Ca	Ti	Fe	Cu	Pb
58.36	5.48	5.64	20.08	0.58	3.29	4.25	0.28	0.57	0.42	1.04

## Abbreviations

The following abbreviations are used in this manuscript:

BFS	blast furnace slag
XPS	X-ray photoelectron spectroscopy
SEM	scanning electron microscopy
EDS	energy dispersive X-ray spectroscopy
XRD	X-ray powder diffraction
AAM	alkali-activated material
HCAAMs	high-calcium alkali-activated materials
LCAAMs	low-calcium alkali-activated materials
